# Do parents modify child-directed signing to emphasize iconicity?

**DOI:** 10.3389/fpsyg.2022.920729

**Published:** 2022-08-25

**Authors:** Paris Gappmayr, Amy M. Lieberman, Jennie Pyers, Naomi K. Caselli

**Affiliations:** ^1^Department of Speech, Language and Hearing Sciences, Boston University, Boston, MA, United States; ^2^Wheelock College of Education and Human Development, Boston University, Boston, MA, United States; ^3^Department of Psychology, Wellesley College, Wellesley, MA, United States

**Keywords:** iconicity, American Sign Language, child-directed language, parent input, sign duration, deafness, language development

## Abstract

Iconic signs are overrepresented in the vocabularies of young deaf children, but it is unclear why. It is possible that iconic signs are easier for children to learn, but it is also possible that adults use iconic signs in child-directed signing in ways that make them more learnable, either by using them more often than less iconic signs or by lengthening them. We analyzed videos of naturalistic play sessions between parents and deaf children (*n* = 24 dyads) aged 9–60  months. To determine whether iconic signs are overrepresented during child-directed signing, we compared the iconicity of actual parent productions to the iconicity of simulated vocabularies designed to estimate chance levels of iconicity. For almost all dyads, parent sign types and tokens were not more iconic than the simulated vocabularies, suggesting that parents do not select more iconic signs during child-directed signing. To determine whether iconic signs are more likely to be lengthened, we ran a linear regression predicting sign duration, and found an interaction between age and iconicity: while parents of younger children produced non-iconic and iconic signs with similar durations, parents of older children produced non-iconic signs with shorter durations than iconic signs. Thus, parents sign more quickly with older children than younger children, and iconic signs appear to resist that reduction in sign length. It is possible that iconic signs are perceptually available longer, and their availability is a candidate hypothesis as to why iconic signs are overrepresented in children’s vocabularies.

## Introduction

All natural human languages–both signed and spoken–contain a range of iconic and arbitrary lexical items (Dingemanse et al., 2015; Winter et al., 2017). In spoken languages, in addition to onomatopoeia, the sounds of words can sometimes reflect aspects of their meanings (e.g., recruiting aspects of the speech signal such as intensity to reference words relating to loudness or excitement). In sign languages, the forms of signs can resemble many aspects of the referent’s size, shape, movement, and texture. Although iconicity is a feature of language across modalities, perhaps due to the affordances of the manual visual modality, it remains more heavily associated with signed languages than with spoken languages (Armstrong and Wilcox, 2007; Meir et al., 2013; Perlman et al., 2018).

### Iconicity and language learning

A growing body of evidence indicates that language learners capitalize on iconicity when learning new lexical items. Adult sign language learners are sensitive to iconic form-meaning mappings (Campbell et al., 1992; Baus et al., 2013), sometimes retaining information about iconicity at the expense of phonology (Ortega and Morgan, 2015). Children, too, are sensitive to iconicity in first language acquisition; parent reports of the vocabularies of deaf signing children show high levels of iconicity, and deaf signing toddlers both comprehend and produce iconic signs more often than non-iconic signs (Thompson et al., 2012, Caselli and Pyers, 2017; Caselli et al., 2017; in BSL: Vinson et al., 2008; in TSL: Sumer et al., 2017). Young children learning spoken languages also show an advantage in learning iconic versus non-iconic words (Imai et al., 2008; Kantartzis et al., 2011; Yoshida, 2012; Imai and Kita, 2014; Perry et al., 2018), and hearing preschoolers learn novel iconic manual symbols more quickly than non-iconic items (Marentette and Nicoladis, 2011; Magid and Pyers, 2017; Ortega et al., 2017). Interestingly, children’s ability to capitalize on the effects of iconicity for word learning seems to interact with their age, with older children learning iconic signs better than younger children (Tolar et al., 2008; Thompson et al., 2012; Magid and Pyers, 2017).

### Learner-centered mechanisms

The mechanisms underlying the effects of iconicity in first language acquisition remain unclear. One set of explanations are what we will term ‘learner-centered’ mechanisms. These appeal to the notion that children are themselves sensitive to iconic mappings and leverage them to learn new words. One example of this kind of theory is Imai and Kita's (2014) sound-symbolism bootstrapping theory, in which children take advantage of an innate ability to map and integrate multi-modal input in order to break into the referential system of language. In essence, sound symbolism bootstraps children’s ability to understand the referential relationship between speech sounds and meaning, which serves as the foundation for building their lexical representations. Similarly, another learner-centered theory might draw upon the structure mapping theory of iconicity (Gentner, 1983; Emmorey, 2014), which suggests that the signer draws an analogy between a mental representation of a concept (e.g., a semantic representation of drinking) and the mental representation of its sign form (e.g., a curved handshape moving to the mouth). In this sort of account, children must have the cognitive capacity to recognize the link between form and meaning.

### Input-centered mechanisms

The other set of explanations for children’s apparent affinity toward iconic signs is ‘input-centered.’ Under this account, adults (either consciously or unconsciously) produce iconic signs in child-directed signing in ways that make these signs more learnable. Patterns in how iconic signs are produced in the input might sufficiently explain most effects of iconicity on acquisition. For example, if iconic signs are used more frequently with children, their frequency alone—and not their iconicity *per se*—might account for their overrepresentation in children’s early vocabularies. Some have hypothesized that child-directed signing may also include the selection of more iconic signs compared to non-iconic signs (Pizer et al., 2011), and in spoken languages, highly iconic (“sound symbolic”) words are more prevalent in child-directed speech than in adult-directed speech (Perry et al., 2015, 2021).

Beyond over-representing iconic signs in their input to children, parents may modify iconic signs during child-directed signing by lengthening, repeating, or enlarging them (Perniss et al., 2018). These differences in how iconic signs are produced are also the characteristics of child-directed signing that are often associated with capturing and maintaining children’s attention (Pizer et al., 2011). Here too, the ways iconic signs are produced may account for their overrepresentation in children’s early vocabularies. Support for this account comes from a longitudinal case study of two Deaf mothers using Israeli Sign Language with their hearing children, reporting that signs were most likely to be repeated, lengthened, enlarged, or displaced (“phonetically modified”) when children are aged 10–14 months, but more likely to be produced with an iconic modification—using iconic mimetic body/mouth/vocal gestures—when children are aged 16–20 months (Fuks, 2020). These results offer early suggestions that parents may systematically produce iconic signs in child-directed interactions in ways that make them easily learned.

### The current study

Learner-centered and input-centered explanations are not mutually exclusive; both forces may be at play in acquisition. Children may leverage their ability to detect iconic mappings to learn new words, and adults may also highlight iconic signs by overrepresenting them in their input and/or modifying them to make them more salient for their children to learn. The current study explores two input-centric ways that child-directed signing might be systematically structured to highlight iconic signs. First, we ask whether parents’ produce iconic signs more often than non-iconic signs with their children, indicating that they are over-representing iconic signs in their interactions with their children. Second, we ask whether parents produce iconic signs with longer durations than non-iconic signs, providing children more time to perceive them, which could in turn make them more learnable. Because the role of iconicity on children’s vocabulary acquisition is impacted by developmental stage, we were most interested to see if these characteristics of iconicity in child-directed signing vary as a function of age. We test these hypotheses by analyzing the use of iconic signs in child-directed signing in a corpus of naturalistic parent–child play interactions in American Sign Language (ASL). The present study is not designed to empirically test any relationships between child-directed signing and child acquisition; rather, by identifying whether iconic signs are highlighted in child-directed signing, we aim to determine whether these input-centered mechanisms are viable hypotheses that account for the advantage of iconicity in child acquisition.

## Materials and methods

### Participants

Participants included 24 parent–child dyads who participated in a naturalistic play session as part of a larger study on ASL development. The children were all deaf and ranged from 9 to 60 months of age (*M* = 36, *SD* = 15). There were 8 females and 16 males. The children’s reported race was White *(n* = 18), Asian (*n* = 1), African American (*n* = 1), more than one race (*n* = 2), or unreported (*n* = 2). Three children had a reported ethnicity as Hispanic/Latinx and 21 as not Hispanic/Latinx. Parents were deaf (*n* = 15) or hearing (*n* = 9), and all parents used ASL to communicate with their deaf child. The interactions were conducted at five sites in the Northeast and Midwest US.

### Data sources

#### ASL-play

The ASL Parent input and Language Acquisition in Young children (ASL-PLAY) dataset is a corpus of naturalistic interactions between parents and their deaf children (Lieberman et al., 2021; Lieberman, 2022). Parents and children were recorded while engaged in a free play interaction. Parents were provided with a standard set of toys including a wooden fruit set, a Lego train set, toy vehicles, and a farmhouse set. Parents were instructed to play as they typically would with their child. Play sessions lasted for approximately 15 min and were recorded from three separate angles to obtain clear views of both the child and parent.

Twelve minutes of each video (beginning one minute after the start of the recording) were coded and analyzed off-line. Videos were coded in ELAN [Crasborn and Sloetjes, 2008; ELAN (Version 5.8), 2019] for a range of features. Signs were glossed individually using the ASL SignBank, a standardized glossing system for ASL (Hochgesang et al., 2020). All signs, English translations, and attention-getters in the ASL-PLAY dataset were annotated using this system by deaf ASL-signing researchers. Signs were tagged individually to capture the onset and offset of each sign. The onset of the sign was defined as the first frame where the sign was identifiable within the sign stream, which typically included the initiation of the movement component of the sign. The offset was the last frame where the sign was still identifiable before transitioning to the next sign.

#### ASL-LEX

ASL-LEX 2.0 is a publicly available online database containing linguistic information for 2,723 ASL signs, selected based on previously published databases, psycholinguistic experiments, and vocabulary tests (Caselli et al., 2017; ASL-LEX 2.0, 2021; Sehyr et al., 2021). It is unclear whether ASL-LEX is representative of the entire lexicon of ASL, and it excludes large pockets of the lexicon (e.g., classifiers); regardless, it is the most comprehensive and only database available. Each sign entry contains detailed lexical and phonological information. Of relevance to this project are the metrics for iconicity, repeated movement, and sign frequency; they are described in detail below. All of the signs in ASL-LEX are cross-referenced with the signs in SignBank, allowing us to merge the lexical data from ASL-LEX with the data from the corpus.

**Iconicity Ratings**: The iconicity estimates in ASL-LEX were derived by averaging over the ratings from 30 hearing non-signers who evaluated how much each sign resembled its meaning (1 = not iconic at all, 7 = very iconic). ASL-LEX also has iconicity ratings from deaf signers for a subset of signs. We chose to use the iconicity ratings from non-signers because ratings from non-signers highly correlate with the ratings from deaf signers (Sehyr and Emmorey, 2019), and were available for the full set of signs in ASL-LEX. The signs in ASL-LEX skew towards being non-iconic, with 66% of signs having an iconicity rating below 4 on a scale of 1–7 (Caselli et al., 2017).

**Repeated Movement**: Each sign in the database is noted as having repeated movement or not. Movement repetition includes repetition of path movements, hand rotation, or handshape change (Sehyr et al., 2021).

**Sign Frequency**: Because there is not a large enough corpus of ASL to robustly estimate lexical frequency, we used the subjective estimates of frequency from ASL-LEX. The frequency estimates in ASL-LEX were averaged over ratings from 25–35 deaf adults who rated how often each sign appears in everyday conversation (1 = very infrequently, 7 = very frequently; Sehyr et al., 2021).

### Data preparation

We extracted all parent sign tokens from participants in the ASL-PLAY dataset (pairs of SignBank Annotation IDs and a timestamp of the duration of the sign in milliseconds), generating a dataset that included 6,294 adult sign tokens from the 24 participants (Per family; *Min* = 68, *Max* = 506, *Mean* = 262).

We identified and removed all point tokens (*n* = 1,256). Points (also called indexes) carry linguistic meaning in ASL; they can serve as pronouns and can also be used to draw attention to an object or event. They were used much more frequently than any other sign; for comparison, the next most common sign type was used 199 times across all parents. Because of their unique linguistic function and the difficulty of assessing their iconicity, we excluded them from the analysis.

We then removed an additional 138 types (*n* = 1,256 tokens) from the dataset consisting of depicting signs, fingerspelled words, gestures, pronouns, idioms, and name signs. These signs did not have an iconicity rating (or a corresponding entry) in ASL-LEX.

Most of the signs in ASL-LEX and SignBank have a 1:1 correspondence, and so can be straightforwardly matched to the ASL-PLAY dataset. Nevertheless, there were some instances in which a sign in the corpus corresponded to two entries in ASL-LEX due to different phonological or inflectional variants (e.g., EAT) with slightly different iconicity ratings; for these cases (*n* = 29 types), we randomly selected one of the two possible matches from ASL-LEX.^1^

The final corpus had 3,782 adult sign tokens representing 371 sign types from 24 participants.

## Results

### Describing parent productions

In order to determine the extent to which each parent favored iconic signs in their signing, we computed a unique mean iconicity rating for each of the 24 parents based on that parent’s sign tokens and types. The total number of tokens per parent ranged from 48 to 318 (*M* = 157, *SD* = 65). Average parent token iconicity ranged from 2.7 to 4.0 (*M* = 3.2, *SD* = 0.3). Parent token iconicity did not differ significantly by parent hearing status (*t* (22) = −0.8 *p* > 0.1). Additionally, there was no relationship between the average iconicity of parent sign tokens and their child’s age (rho = 0.03, *p*= > 0.1). Number of parent sign types ranged from 23–103 (*M* = 57, *SD* = 21), and the average iconicity of those sign types ranged from 2.7–3.6 (*M* = 3.2, *SD* = 0.2). Across all family tokens, the distribution of parent sign tokens by lexical category (taken from ASL-LEX), was as follows: 1125 nouns (30%), 1,090 verbs (29%), 778 minor class items (21%), 455 adjectives (12%), 282 adverbs (7%), and 52 numbers (1%). A table summarizing the participant data from all 24 families is included in the Appendix.

### Iconicity of child-directed signs relative to ASL-LEX

**Figure 1:**
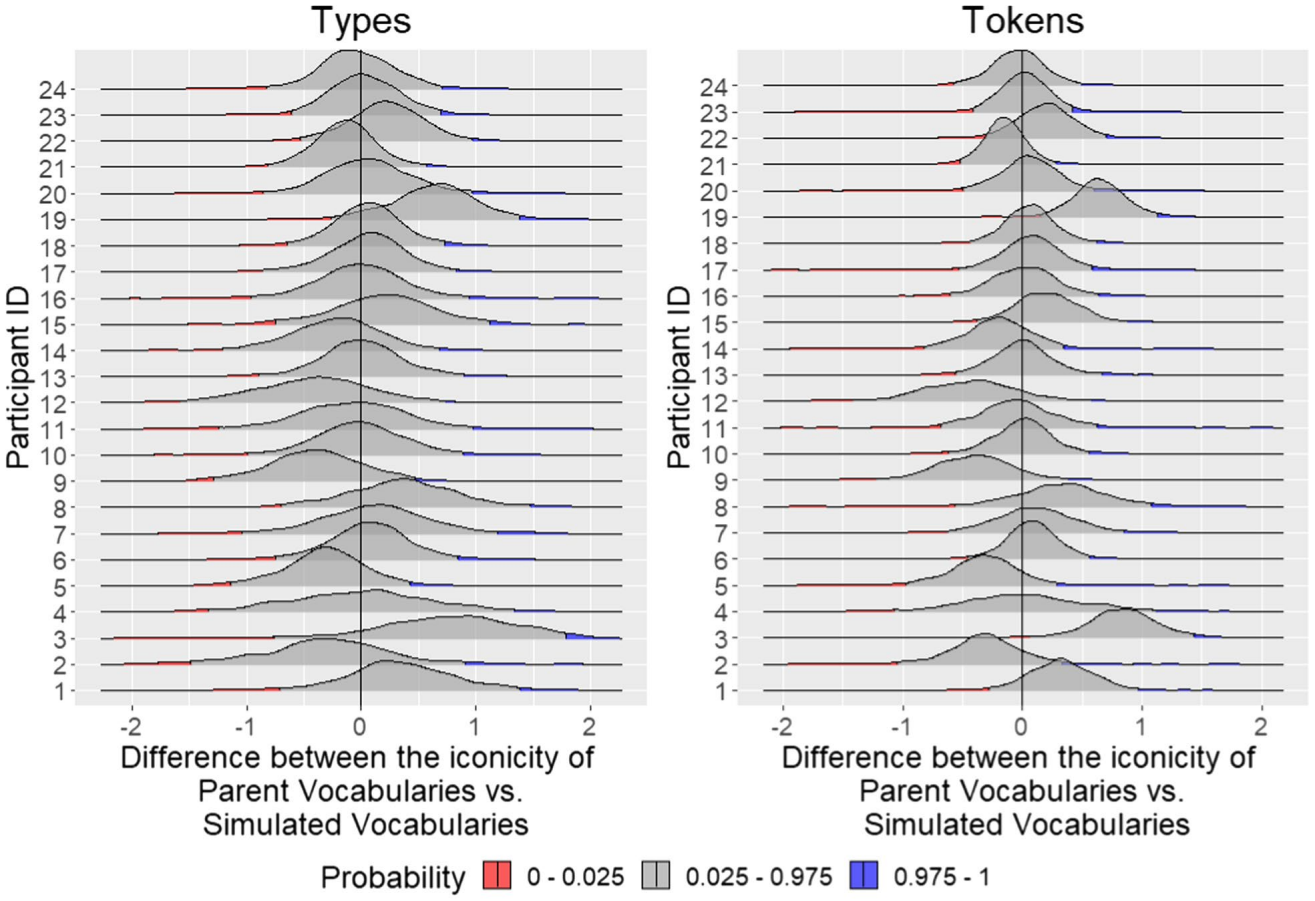
The distribution of difference in mean iconicity of 1,000 pairs of Parent Vocabularies and Simulated Vocabularies. The upper and lower bounds of the 95% confidence interval are illustrated in blue and red, respectively. Distributions that largely fall above zero (i.e., the lower bound of the 95% CI is above 0) indicate that parents’ signs were more iconic than chance. In the left panel, iconicity ratings were averaged over sign types, and in the right panel over sign tokens. With the exception of two parents’ tokens (participants 3 and 19), Parent Vocabularies were no more iconic than would be expected by chance.

We first asked whether parents’ child-directed signs were more iconic than one might expect by chance. To do this, we compared bootstrapped estimates of the iconicity of the sign types the parents actually used with their children during the session (Parent Vocabularies) to simulated vocabularies of the same number of items randomly drawn from the ASL-LEX database (Simulated Vocabularies) to represent the “lexicon” of each parent during the play session. We also conducted a parallel analysis of sign tokens by comparing all individual tokens the parents produced with their children to simulated vocabularies with the same number of items randomly drawn from ASL-LEX, but with replacement so the same item could appear more than once to account for individual token productions. To control for lexical frequency in the simulated vocabularies, for both tokens and types, the random samples from ASL-LEX were weighted by frequency. The simulated vocabularies were designed to estimate how iconic a set of signs might be by chance. We bootstrapped Parent Vocabularies by randomly sampling from a subset of either tokens or types from each parent’s attested items, calculated the mean iconicity rating of each subsample, and repeated this process 1,000 times. We then paired one Simulated Vocabulary with one Parent Vocabulary and calculated the difference in mean iconicity of each vocabulary. We visualized the distribution of the 1,000 difference scores for each of the 24 parents in Figure 1.

If parents’ vocabularies were significantly more iconic than chance, we would expect the difference between the bootstrapped Parent Vocabularies and the Simulated Vocabularies to be significantly larger than zero (i.e., 0 should fall below the 95% CI). Instead, what we found is that for both tokens and types, the mean iconicity of the bootstrapped Parent Vocabularies is comparable to the Simulated Vocabularies. For sign types, the iconicity estimates of all the Parent Vocabularies were indistinguishable from zero. The same is largely true of the tokens, though two parents used iconic signs more often than chance (probability <0.025), suggesting that those two parents may systematically repeat iconic signs (Figure 1). Contrary to our predictions, iconic signs were not overrepresented in child-directed signing.

### What factors predict sign duration in parent input?

We next sought to determine whether more-iconic signs were produced with longer duration relative to less-iconic signs. We ran a linear mixed-effect model to determine whether iconicity of parent sign productions predicted their duration. The dependent variable was token duration. The critical predictor was an interaction between iconicity and age. Two other control variables that may influence duration were drawn from ASL-LEX: (1) repeated movement, since signs that had repetition would take physically longer to produce, and (2) sign frequency. We included sign frequency because it is often inversely related to phonetic duration, as seen across spoken languages (e.g., Gahl et al., 2012), and in Swedish Sign Language (Börstell et al., 2016). Finally, the model included parent hearing status and random effects for participants (Table 1).

**Table 1:** Results of the model predicting sign duration.

Predictors	Adult sign duration		
	Estimates	CI	p
(Intercept)	1.05	0.85–1.25	<0.001
Iconicity	−0.03	−0.05–0.00	0.088
Repeated movement	0.10	0.06–0.13	<0.001
Frequency in lexicon	−0.08	−0.10– −0.06	<0.001
Child age	−0.00	−0.01– −0.00	0.064
Parent hearing status	−0.03	−0.17–0.10	0.626
Iconicity x Age	0.00	0.00–0.00	0.038

There were significant positive effects of repeated movement, and significant negative effects of sign frequency, child age, and the interaction between iconicity and age.

**Figure 2:**
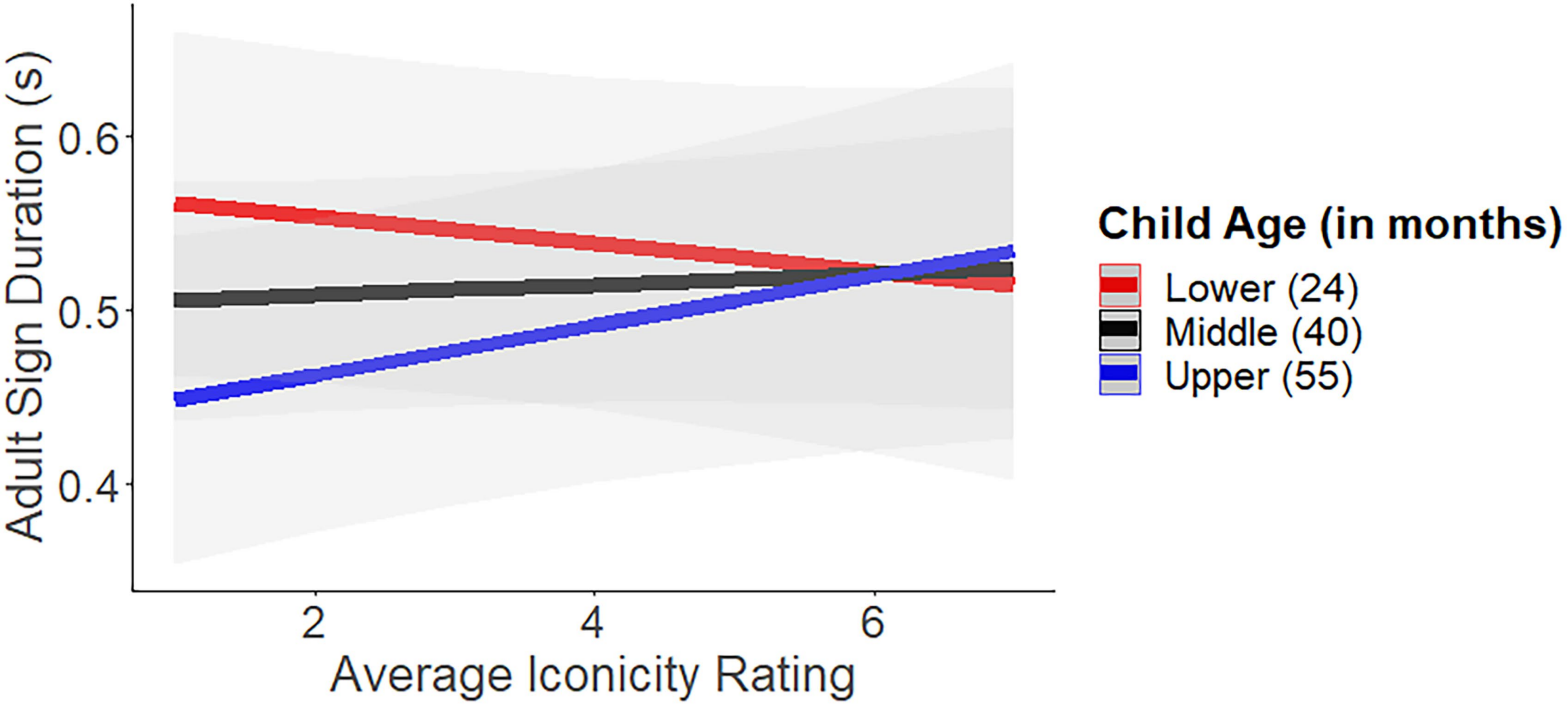
The interaction between sign duration, iconicity and child age in months. For younger and middle-aged children sign duration was similar regardless of the sign’s iconicity rating, but for older children sign duration was shorter for non-iconic signs than iconic signs. The lines indicate the children’s mean age and +/− one standard deviation.

In support of the hypothesis, there was an interaction between iconicity and age. Visualization of the model (Figure 2) illustrates that parents of younger children had similar sign durations for iconic and non-iconic signs, but parents of older children had shorter durations for non-iconic signs. Simple slopes analyses confirmed this pattern; the only slope that was marginally different from zero was that of the oldest children (*B* = 0.02(3612.4), *SE* = 0.008, *p* = 0.053). Notably, for the older children, the parents’ iconic signs had similar durations to those of the parents’ of the younger children. This finding provides weak evidence that parents may begin to shorten non-iconic signs as their children get older, but that iconic signs seem to resist shortening.

## Discussion

We examined a corpus of parent interactions with deaf children to investigate iconicity in child-directed signing. First, we found that the average iconicity of parent productions were largely no different than chance (i.e., than the average iconicity of a random sample of signs drawn from the larger ASL lexicon). Only two of the 24 parents produced sign tokens that were more iconic than expected by chance. This pattern suggests that the frequency of iconic signs in child-directed signing is an unlikely explanation for the previously documented advantage for iconic signs in children’s vocabularies. Second, we found patterns in our data suggesting that sign duration in child-directed signing may be systematically different for highly iconic and less iconic signs as a function of age: while parents of younger children had similar sign durations for both low and high iconicity signs, parents of older children had shorter duration for low iconicity signs than high iconicity signs. If this pattern holds in future studies, we would take it to indicate that the duration of the iconic signs stays constant as children grow. That is, while parents shorten the articulation of low iconicity signs, iconic signs resist this reduction, leading to increased salience of iconic signs in the input and a corresponding advantage in the acquisition of these signs.

### Prevalence of iconic lexical items in parent input

The fact that parents did not overrepresent iconic signs when signing with their children is somewhat different from previous work on use of iconic words in child-directed speech; Perry et al. (2018) found that parent–child conversations use highly iconic words more frequently than adult conversations. This difference may be methodological: the children in our sample had a wider age range and were, on average, older than those in Perry et al. (2018), and the toys available for dyads to play with during the present play sessions may not have elicited especially iconic signs. Alternatively, it could be that there are modality differences in child-directed language in signed vs. spoken languages. Sign languages are more iconic overall than spoken English (Dingemanse et al., 2015; Perlman et al., 2018), and so inflating the rates of iconicity may not be natural to parents; since the language already makes use of iconic form-meaning mappings, inflating those iconic mappings further might not be intuitive.

### Differential modification of iconic signs

We found that the duration of iconic signs varies systematically in children’s input, whereby parents produce iconic signs for longer than less iconic signs, but this effect depends on age. With the youngest children in our sample, parents did not vary their sign duration as a function of degree of iconicity. For the older children in our sample (age four years and up) parents produced iconic signs for longer than less iconic signs. This finding aligns with prior literature on modifications of child-directed signing (Perniss et al., 2018), and with studies showing that the effect of iconicity on children’s acquisition is greatest among older hearing children (aged 3+; Namy et al., 2004; Tolar et al., 2008) rather than younger ones (aged 18–24 months; Perry et al., 2021).

However, much of the research concerning iconicity in early sign language acquisition targets children within the first 20 months (10–14 months- Massaro and Perlman, 2017; 21–30 months- Thompson et al., 2012). While the older children in the current study may see iconic signs for longer, they may have already acquired those signs. So, the function of parents’ lengthening of iconic signs in their child directed signing to older children remains unclear.

There are two ways to consider the observed interaction between iconicity and age on sign duration: parents may lengthen iconic signs or reduce non-iconic signs. Because the length of iconic signs is similar for parents of younger and older children, our interpretation is that iconic signs resist reduction. Lengthening is a common property of child-directed signing (e.g., Holzrichter and Meier, 2000; Pizer et al., 2011), and as children grow parents typically produce signs more rapidly. This study suggests that iconic signs resist this shortening of sign duration and remain similar in length to the input much younger children receive.

While the present study is not designed to determine whether increased sign duration causes children to more readily learn signs, it suggests that an ‘input centric’ mechanism is a viable explanation as to why iconic signs are overrepresented in older children’s early vocabularies: iconic signs are perceptually available for longer, which may make them easier for children to learn. Another mutually compatible possibility is that parents lengthen iconic signs *in response* to children’s acquisition, lengthening these signs because they are aware that children are learning them. More work is needed to identify the nature of the relationship between the lengthening of iconic signs in child-directed signing and acquisition of those signs.

### The role of visual attention

We speculate that children’s ability to monitor and manage their own visual attention may partially explain the influence of child age on parent sign duration. Specifically, older children are better able to control their visual attention, so they are more likely to be looking at their parents when signs are produced. Pizer et al. (2011) found a significant association between child eye gaze and parent sign duration, with parents producing longer signs when they did not have eye contact with their child. It is likely that children in the current study were old enough to skillfully manage their own attention, resulting in parents producing shorter signs overall but maintaining the increased length of iconic signs due to their phonological form or other factors. Future studies that take into account children’s eye gaze to the parent during interaction will help shed light on this possibility.

### Limitations and future directions

Our analysis looked only at lexicalized signs which had a corresponding entry in ASL-LEX that included an iconicity rating. Depicting signs show appearance, location, and/or movement- are often transparently iconic, but were excluded from analysis here. In addition to the iconicity of the manual components of depicting signs, signers often produce accompanying mouth movements that are temporally aligned with the production of the sign and depict the referent’s size and shape in iconic ways (Lu and Goldin-Meadow, 2018). Importantly, if lexical signs do not map neatly onto their referents, depicting signs may be used instead to better align with an iconic mapping (Lu and Goldin-Meadow, 2018), which may increase the overall iconic properties of child-directed signing, even within our corpus. How iconicity influences parents’ production of depicting signs may very well be different from the lexical items in this study, and merits further exploration.

In the current study we investigated the hypothesis that the sign duration of iconic signs may be longer than non-iconic signs. In addition to lengthening, parents may specifically highlight iconic signs by repeating them, displacing them into the child’s view, using an unconventional place of articulation, or even attempting to explain the iconic properties of the sign (e.g., Pizer et al., 2011). Perniss et al. (2018) found that parents modify iconic signs more than non-iconic signs, particularly in non-ostensive naming contexts. While these findings support our work, it is important to note that all our contexts were ostensive, with the toys present throughout the interaction, which may have impacted the likelihood of iconic signs being lengthened. Though Perniss et al. do not report the proportion of each kind of modification in their study (enlargement, repetition, and lengthening), Fuks (2020) found that when signs were phonetically modified, they were most likely to be repeated or enlarged, not lengthened. Seeing as our study did not analyze other forms of modification, iconic signs may have been emphasized in other ways within the corpus. Moreover, the kind of modification that parents apply to iconic signs may specifically illustrate the iconicity of the sign. For example, signs referencing large objects might be more likely to be enlarged, signs referencing slow objects might be more likely to be lengthened, etc. Signs can be iconic of their referent in a myriad of ways, and parents can highlight that iconicity by using many forms of modification. More research is needed to examine these other ways that iconic signs may be modified in child-directed signing, especially in naturalistic contexts.

## Conclusion

This study of parent input during naturalistic ASL interactions revealed that parents do not preferentially use iconic signs, but may lengthen their sign productions as a function of iconicity for older children. Increased sign duration may support children’s acquisition of iconic signs, but more work is needed to determine whether there is a causal relationship between the length of iconic signs in input and their acquisition. Though we find effects of iconicity in child-directed signing, the effects were subtle. Thus, we await a more nuanced analysis of other types of sign modifications to better understand how input-centered mechanisms might relate to the acquisition of iconic signs. The current study contributes to our understanding of how iconic signs are produced in child-directed signing, and lays groundwork for investigations of the relationship between child-directed signing and child vocabulary acquisition.

## Data availability statement

The data that support the findings of this study are available upon reasonable request from the corresponding author.

## Ethics statement

The studies involving human participants were reviewed and approved by the Boston University IRB. Written informed consent to participate in this study was provided by the participants' legal guardian/next of kin.

## Author contributions

PG conceptualized the study, prepared the data, and conducted the analyses. AL helped to collect the data for the ASL-PLAY dataset and contributed to data analysis. NC collected the data for the ASL-LEX database and contributed to data analysis. JP contributed to data analysis. All authors contributed to writing the manuscript and approved the submitted version.

## Funding

This research was supported by the National Institute on Deafness and Other Communication Disorders (Award Nos. DC015272 and DC018279), as well as the National Science Foundation (Award Nos. BCS-1918252 and BCS-1625793).

## Conflict of interest

The authors declare that the research was conducted in the absence of any commercial or financial relationships that could be construed as a potential conflict of interest.

## Publisher’s note

All claims expressed in this article are solely those of the authors and do not necessarily represent those of their affiliated organizations, or those of the publisher, the editors and the reviewers. Any product that may be evaluated in this article, or claim that may be made by its manufacturer, is not guaranteed or endorsed by the publisher.

## Footnotes

^1^To ensure that this approach did not unduly influence the analysis, we repeated a parallel set of analysis in which we selected the highest of the two iconicity ratings for each item rather than a random selection. The results were qualitatively the same.

## Appendix

**Table A1:** Summary of dataset.

ID #	Child age in months	Parent hearing status	# of raw tokens produced	# of tokens included in analysis	# of sign types	Average iconicity	Average sign duration	Number of nouns	Number of verbs
1	9	Hearing	244	149	43	3.44	0.58	35	45
2	12	Hearing	181	118	32	2.82	0.5	55	28
3	16	Hearing	275	164	33	3.96	0.49	45	56
4	20	Hearing	68	48	23	3.15	0.63	19	19
5	25	Deaf	215	129	60	2.82	0.6	40	29
6	25	Deaf	332	210	68	3.21	0.31	56	43
7	28	Deaf	153	103	47	3.25	0.49	35	39
8	29	Deaf	128	70	34	3.48	0.34	12	24
9	29	Hearing	113	82	39	2.72	0.48	20	12
10	32	Hearing	284	188	60	3.12	0.53	81	51
11	33	Hearing	152	109	42	3.07	0.6	25	37
12	33	Deaf	89	54	28	2.69	0.88	17	18
13	34	Deaf	260	146	63	3.12	0.38	54	36
14	35	Hearing	235	158	63	2.93	0.6	69	23
15	35	Deaf	269	131	44	3.33	0.45	33	26
16	41	Deaf	216	131	60	3.13	0.6	35	39
17	38	Deaf	290	182	69	3.19	0.35	36	58
18	42	Deaf	397	222	80	3.21	0.45	59	33
19	47	Deaf	400	244	67	3.76	0.51	62	85
20	56	Deaf	360	206	79	3.18	0.61	69	44
21	59	Deaf	426	318	103	2.98	0.25	83	85
22	59	Deaf	340	197	84	3.33	0.22	67	54
23	59	Deaf	506	238	85	3.14	0.29	40	65
24	60	Hearing	361	185	80	3.09	0.6	48	55
